# Targeted Sequencing of 242 Clinically Important Genes in the Russian Population From the Ivanovo Region

**DOI:** 10.3389/fgene.2021.709419

**Published:** 2021-10-07

**Authors:** Vasily E. Ramensky, Alexandra I. Ershova, Marija Zaicenoka, Anna V. Kiseleva, Anastasia A. Zharikova, Yuri V. Vyatkin, Evgeniia A. Sotnikova, Irina A. Efimova, Mikhail G. Divashuk, Olga V. Kurilova, Olga P. Skirko, Galina A. Muromtseva, Olga A. Belova, Svetlana A. Rachkova, Maria S. Pokrovskaya, Svetlana A. Shalnova, Alexey N. Meshkov, Oxana M. Drapkina

**Affiliations:** ^1^National Medical Research Center for Therapy and Preventive Medicine, Moscow, Russia; ^2^Faculty of Bioengineering and Bioinformatics, Lomonosov Moscow State University, Moscow, Russia; ^3^Moscow Institute of Physics and Technology, Dolgoprudny, Moscow, Russia; ^4^Novosibirsk State University, Novosibirsk, Russia; ^5^All-Russia Research Institute of Agricultural Biotechnology, Moscow, Russia; ^6^Cardiology Dispensary, Ivanovo, Russia

**Keywords:** genetic testing, rare variants, secondary findings, pathogenic variants, penetrance

## Abstract

We performed a targeted sequencing of 242 clinically important genes mostly associated with cardiovascular diseases in a representative population sample of 1,658 individuals from the Ivanovo region northeast of Moscow. Approximately 11% of 11,876 detected variants were not found in the Single Nucleotide Polymorphism Database (dbSNP) or reported earlier in the Russian population. Most novel variants were singletons and doubletons in our sample, and virtually no novel alleles presumably specific for the Russian population were able to reach the frequencies above 0.1–0.2%. The overwhelming majority (99.3%) of variants detected in this study in three or more copies were shared with other populations. We found two dominant and seven recessive known pathogenic variants with allele frequencies significantly increased compared to those in the gnomAD non-Finnish Europeans. Of the 242 targeted genes, 28 were in the list of 59 genes for which the American College of Medical Genetics and Genomics (ACMG) recommended the reporting of incidental findings. Based on the number of variants detected in the sequenced subset of ACMG59 genes, we approximated the prevalence of known pathogenic and novel or rare protein-truncating variants in the complete set of ACMG59 genes in the Ivanovo population at 1.4 and 2.8%, respectively. We analyzed the available clinical data and observed the incomplete penetrance of known pathogenic variants in the 28 ACMG59 genes: only 1 individual out of 12 with such variants had the phenotype most likely related to the variant. When known pathogenic and novel or rare protein-truncating variants were considered together, the overall rate of confirmed phenotypes was about 19%, with maximum in the subset of novel protein-truncating variants. We report three novel protein truncating variants in *APOB* and one in *MYH7* observed in individuals with hypobetalipoproteinemia and hypertrophic cardiomyopathy, respectively. Our results provide a valuable reference for the clinical interpretation of gene sequencing in Russian and other populations.

## Introduction

The next-generation sequencing projects of the last decade revealed the complicated spectrum of rare and ultra-rare allelic variation in most human genes (Tennessen et al., 2012; Lek et al., 2016; Van Hout et al., 2020). The sequencing of large population cohorts greatly enriched our understanding of the prevalence of presumably pathogenic variants in reportedly healthy people (Dorschner et al., 2013; Amendola et al., 2015; Dewey et al., 2016) and initiated studies aimed at reviewing the implied pathogenicity of genetic variants (Dewey et al., 2014; Shah et al., 2018) and penetrance of variants classified as pathogenic (Cooper et al., 2013; Chen et al., 2016; Wright et al., 2019; Van Rooij et al., 2020). This activity was shaped by the standards and guidelines for the classification of sequence variants using criteria informed by expert opinion developed by The American College of Medical Genetics and Genomics (ACMG) (Richards et al., 2015; Nykamp et al., 2017).

The ACMG also suggested the list of genes for which the reporting of known and expected pathogenic variants, also termed incidental findings, may be recommended (Green et al., 2013). The updated guidelines presented ACMG59, the list of the mostly dominant 59 genes associated with medically actionable disorders (Kalia et al., 2017). However, limited information on the penetrance of many variants even in this relatively small set of genes makes interpretation challenging. This uncertainty is gradually decreasing as more studies evaluate the carrier status of pathogenic gene variants in general populations (Amendola et al., 2016; Wright et al., 2019; Van Rooij et al., 2020). Numerous papers reported the prevalence of known and expected pathogenic variants in various populations, for example, Dutch (Haer-Wigman et al., 2019; Van Rooij et al., 2020), Qatari (Jain et al., 2018), Korean (Kwak et al., 2017), Australian (Lacaze et al., 2020), British (Van Hout et al., 2020), and Taiwanese (Kuo et al., 2020). The present study investigates the incidental findings of pathogenic variants in 28 genes from the ACMG59 list in the Russian population from the Ivanovo region.

Russia is one of the most ethnically diverse countries in the world; however, it is underrepresented in the large human genome sequencing projects (Lek et al., 2016; Van Hout et al., 2020). Earlier genome-wide studies of the Russian population were mostly based on the limited number of samples and focused on the genetic history and admixture patterns (Mallick et al., 2016; Wong et al., 2017). Recently, the whole-genome variation was analyzed for the 264 healthy adult participants of the Genome Russia Project (Zhernakova et al., 2020) demonstrating adaptive, clinical, and functional consequences. Approximately 3–4% of the called variants were classified as novel when compared to the dbSNP database (Sherry et al., 2001); in many cases, allele frequencies demonstrated divergence from the neighboring populations. Barbitoff et al. (2019) reported variant frequencies in a larger subset of 694 exomes from the northwest Russia. The results indicated that 9.3% of discovered variants were not present in dbSNP. Moreover, this whole-exome study demonstrated the overrepresentation of several disease-causing variants for Mendelian disorders, such as phenylketonuria (*PAH*, rs5030858), Wilson’s disease (*ATP7B*, rs76151636), factor VII deficiency (*F7*, rs36209567), and the kyphoscoliosis type of Ehlers–Danlos syndrome (*FKBP14*, rs542489955). For the Russian population, however, pathogenic variant frequencies were reported mostly for relatively small cohorts including patients and their families and targeted at specific genes and disorders, for example, familial hypercholesterolemia (Meshkov et al., 2021; Miroshnikova et al., 2021); cystic fibrosis, phenylketonuria, alpha-1 antitrypsin deficiency, and sensorineural hearing loss (Kiseleva et al., 2020; Petrova et al., 2020); cardiomyopathy (Marakhonov et al., 2019; Zaklyazminskaya et al., 2019; Kulikova et al., 2021; Shestak et al., 2021); and breast and ovarian cancer (Brovkina et al., 2018; Solodskikh et al., 2019).

The aim of this work was to take a step forward in the study of the genetic makeup of the Russian population, with the emphasis on rare variation, in particular, known and expected pathogenic variants in a subset of clinically important genes. We developed the gene panel which included 242 protein-coding genes associated with cardiovascular diseases and high risk of early or sudden cardiac death and used the population samples collected in the course of the ESSE-RF cardiovascular epidemiology project (Boitsov et al., 2013). Below, we report the results of the targeted sequencing of 1,685 unrelated participants from Ivanovo, one of the ESSE-RF regions. The availability of clinical data made it possible to evaluate the carriers of known and expected novel pathogenic variants for certain phenotypes.

## Materials and Methods

### Selection of Participants and Clinical Data

The individuals for our study were selected from the study “Epidemiology of Cardiovascular Diseases and Risk Factors in Regions of the Russian Federation (ESSE-RF).” The ESSE-RF is a multicenter population-based study, conducted from 2012 to 2014, covering 13 regions of Russia, differing in climatic, geographic, economic, and demographic characteristics (Boitsov et al., 2013). About 1,600–1,900 people, aged 25–64, were randomly selected from every region, including Ivanovo, a region approximately 300 km northeast of Moscow with predominant Russian population. Please see more details regarding sample selection in the Supplementary Methods.

The sequenced set contained 1,685 individuals: 1,056 females with median age 52 at the moment of enrollment and 629 males with median age 44, respectively. We used PLINK v1.90 (Chang et al., 2015) to calculate identity by state (IBS) values and estimate the identity by descent (IBD) proportion (PI_HAT) for all possible pairs of individuals. To ensure that our dataset does not include closely related participants, we removed all pairs with PI_HAT > 0.33. The average PI_HAT value across all 1,418,770 pairs of 1,685 individuals was 0.027.

Clinical data were obtained from several major sources: (1) questionnaires administered face-to-face at the beginning of the ESSE-RF study (2012), (2) fasting venous blood samples, (3) electrocardiogram (ECG) records, (4) coronary artery disease (CAD) validation in 2013, and (5) endpoint data. Additionally, (6) the available medical records of 10 patients with variants of interest in the ACMG59 genes observed at the Ivanovo Cardiology dispensary were analyzed. Blood samples for genetic analysis were stored at the Biobank of the National Medical Research Center for Therapy and Preventive Medicine (Moscow, Russia) (Pokrovskaya et al., 2019; Anisimov et al., 2021). Demographic characteristics, information about the most common non-communicable diseases, ECG parameters, and multiple blood biochemical parameters including lipid profiles are included into analysis. Endpoints such as all-cause mortality, myocardial infarctions, strokes, new cases of coronary artery disease, and revascularization are collected annually in the ESSE-RF. Clinical and endpoint data were available for all patients. Coronary artery disease was verified for participants with positive Rose Angina Questionnaire or the patient’s positive answer to questions concerning previous diagnoses of either coronary artery disease or myocardial infarction. It is worth noting, however, that even with these data at hand, we were able to evaluate the individuals with known or expected pathogenic mutations for a limited number of phenotypes, for example, hypobetalipoproteinemia and prolonged QT interval.

### Ethics Statement

The study was reviewed and approved by the Independent Ethic Committee of the National Medical Research Center for Therapy and Preventive Medicine (Protocol number 07-03/12 from 03.07.2012) and conducted according to the principles of the Helsinki Declaration. The participants provided their written informed consent to take part in this study.

### Target Panel Design and Sequencing

We developed the targeted panel including coding exons of 242 protein-coding genes associated with cardiovascular diseases and high risk of early or sudden cardiac death, in particular, cardiomyopathy (for example, *MYBPC3*, *MYH7*, *DSP*, *LMNA*, and *DES*), channelopathy (*KCNQ1*, *KCNH2, SCN5A*, and others), and hypercholesterolemia and hypobetalipoproteinemia (*APOB*, *PCSK9*, *LDLR*, *LDLRAP1*, *ANGPTL3*, and others). Exon coordinates were expanded by 25 bp to include splice region variants. The sequenced target contained 4,311 exons with the total length of 1.04 Mbp. The following 28 genes from our panel are in the list of 59 well-characterized medically actionable disease genes recommended by the American College of Medical Genetics and Genomics for the return of incidental findings in clinical genomic sequencing (Kalia et al., 2017): *ACTA2*, *ACTC1*, *APOB*, *DSC2*, *DSG2*, *DSP*, *GLA*, *KCNH2*, *KCNQ1*, *LDLR*, *LMNA*, *MYBPC3*, *MYH11*, *MYH7*, *MYL2*, *MYL3*, *PCSK9*, *PKP2*, *PRKAG2*, *RYR1*, *RYR2*, *SCN5A*, *SDHD*, *SMAD3*, *TMEM43*, *TNNI3*, *TNNT2*, and *TPM1*. These genes comprise the cardiovascular disorder-related part of the ACMG59 panel. We refer to this subset of our target as ACMG59 genes. The full list of targeted genes with phenotypes is shown in Supplementary Table 1.

### Sequencing Data Analysis and Variant Annotation

Target sequencing data processing and quality control evaluation were performed with the custom- designed pipeline based on GATK 3.8 and generally following the Broad institute best practices for variant calling with both standard GATK hard filters and VQSR (details in the Supplementary Methods). Paired-end reads were aligned to the hg19 genome sequence which is the major human reference in this project. Since the VQSR quality evaluation is more effective for the hg38, we also ran the pipeline with this reference and used the resulting filters independently: a variant was accepted for downstream analysis only if both hg37 and hg38 GATK filters flagged it as PASS and all non-reference genotype quality values were maximal (GQ = 99). This filtering retained 11,876 (84.3%) variants of the initial set of 14,087 variants and is a conservative approach aimed at minimizing the false positive variant calls.

Single-nucleotide variants and short indels were annotated with ENSEMBL Variant Effect Predictor (VEP) v.100 (McLaren et al., 2016) and cross-checked against the standalone versions of ClinVar (2021/01/10) and gnomAD (v2.1.1) databases that accumulate information on variant clinical significance and population frequencies (Landrum et al., 2016; Karczewski et al., 2020). We used ClinVar as the major source of known pathogenic and likely pathogenic variants (KP). VEP identified known variants present in the dbSNP database (Sherry et al., 2001). For each annotated variant, VEP aggregates maximal alternative allele frequency across 1000 Genomes, ESP and ExAC/gnomAD, and reports in the MAX_AF field. Allele frequencies in our dataset were also compared, where available, to those in the Russian northwest population (NWR) (Barbitoff et al., 2019).

We used LOFTEE (Karczewski et al., 2020), SIFT (Ng and Henikoff, 2006), and PolyPhen-2 (Adzhubei et al., 2010) VEP plugins to flag low-confidence protein-truncating variants (PTVs, high- impact variants in VEP) and predict potentially damaging missense variants, respectively. Missense variants simultaneously predicted as deleterious by SIFT and damaging by PolyPhen-2 are referred to as strictly damaging. We used VarSome (Kopanos et al., 2019) to perform automated variant classification according to the ACMG guidelines (Richards et al., 2015).

## Results

### Novel Variants

We explored the spectrum of genome variation in the Russian population by sequencing 242 clinically important protein-coding genes in 1,658 unrelated individuals from the Ivanovo region. The strict quality control and filtering procedure described in the section “Materials and Methods” filtered out 2,209 variants out of the initial 14,087 keeping 11,423 SNVs and 453 short indels in the targeted regions. The observed transition/transversion Ti/Tv ratio was 3.10 for all SNVs in the exons and flanking regions and 3.48 for missense and synonymous variants suggesting the paucity of false positive variants after filtering.

**FIGURE 1:**
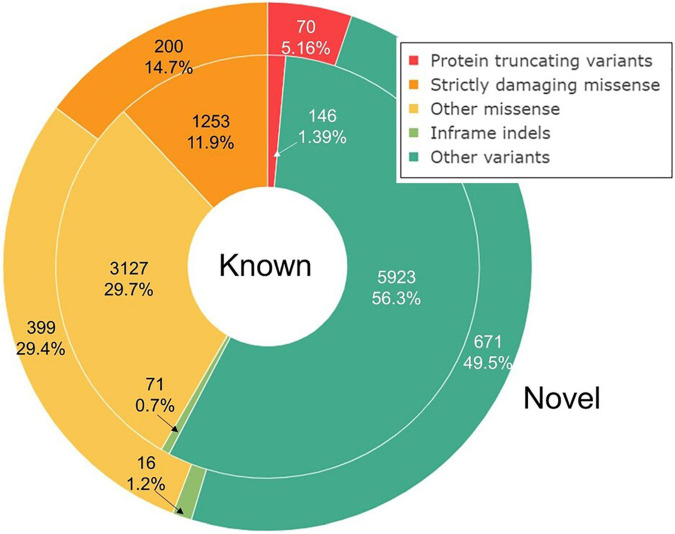
Variant annotation. Known and novel variants are represented by inner and outer rings, respectively. Variant annotation was performed by ENSEMBL VEP. Strictly damaging variants are those predicted as damaging both by SIFT and PolyPhen-2.

**FIGURE 2:**
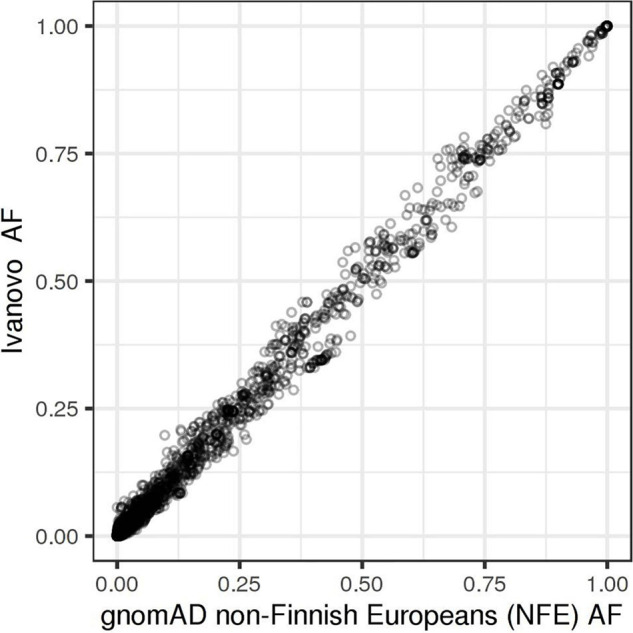
Allele frequencies in the Ivanovo population (y-axis) compared to gnomAD non-Finnish Europeans (x-axis).

Missense variants comprise the largest group by annotation (40.8%), followed by synonymous (27.2%) and intron variants (21.3%). We split the bulk of the observed variation into five groups based on their annotation and potential impact on gene function: protein-truncating variants (PTVs), strictly damaging missense variants (predicted to be damaging both by SIFT and PolyPhen-2), other missense variants, in-frame indels, and other variants, including synonymous, UTR, intron, and other variants with the lowest expected impact (Table 1 and Figure 1). The fraction of strictly damaging predictions is maximal among singleton missense variants (31.9%) and gradually reduces with increasing frequency, reaching 15.1% for missense variants with allele frequency in our sample AF > 1%. This confirms the observation of a negative correlation between the alternative allele frequency and the ratio of missense variants predicted as damaging in large cohorts (Tennessen et al., 2012). Out of the total 11,876 high-quality SNV and short indels discovered in our dataset, 7,582 (63.8%) were singletons or doubletons (allele count AC < 3) with estimated alternative allele frequency not exceeding approximately 0.1%. The overwhelming majority (99.3%) of variants detected in our study in three or more copies are reported in the dbSNP and shared with other populations. The allele frequencies of these variants are very close to those in the European non-Finnish (NFE) gnomAD exomes with Pearson’s correlation coefficient *R* = 0.997 (Figure 2).

**TABLE 1:** Overview of variants in the 242 targeted genes in the Ivanovo population.

	**Allele count AC < 3**		**Allele count AC ≥ 3**	
	**Known**	**Novel (Not in NWR)**	**Known**	**Novel (Not in NWR)**
Protein truncating variants	112	70 (69)	34	0 (0)
Strictly damaging missense variants	907	193 (190)	346	7 (5)
Other missense	1,957	395 (379)	1,170	4 (4)
In-frame indels	49	15 (15)	22	1 (1)
Other variants	3,227	657 (635)	2,696	14 (3)
Total	6,252	1,330	4,268	26

*Novel: not in dbSNP 153.*

*NWR: 694 exomes from the northwest Russia (Barbitoff et al., 2019).*

*AC, allele count.*

A total of 1,356 (11.4%) variants out of 11,876 were novel, that is, not present in dbSNP 153. The fraction of novel variants was highest among the protein-truncating ones (32.4%) apparently due to the purifying selection acting on specific types of variants enriched with deleterious alleles (Lek et al., 2016). In-frame indels were not that abundant but include the second largest fraction of novel variants (18.4%). The majority (95.9%) of novel variants were not reported earlier in the survey of 694 exomes from the northwest Russia (NWR) (Barbitoff et al., 2019), the largest report on the whole exome sequencing in the Russian population to date. This is not surprising, taking in view the fact that our set is 2.4 times larger and thus allows the surveying of a wider spectrum of rare variation.

The novel variants in the Ivanovo population were found mostly among singletons and doubletons with only 26 variants with three-to-seven copies of alternative alleles (Supplementary Table 2). There were no novel protein-truncating variants other than singletons or doubletons. In Table 2, we report 12 selected novel variants with three or more alleles in our dataset and that were annotated as moderate impact by VEP. All these variants were missense except for a complex non-frameshifting variant in *TRIM63* that was reported by GATK as three adjacent short indels. This complex haplotype was confirmed by the visual inspection of read alignments with IGV software (Robinson et al., 2011) and reported in Table 2 as a single in-frame indel spanning protein residues 98–109. Seven missense variants were predicted as damaging both by SIFT and PolyPhen-2 and were denoted with asterisk in Table 2. The remarkably large *SYNE2* gene harbored two such novel strictly damaging variants, Thr3804Asn and Glu4972Lys, each observed in three distinct individuals. Only two variants (Ser291Phe in *DMD* and Lys4037Glu in *SYNE1*) were found earlier in NWR exomes. The relatively high prevalence of these variants in the Russian population should be taken into account in the course of exome or genome sequencing in the clinical context (Richards et al., 2015).

**TABLE 2:** Novel variants with three or more alleles in our dataset annotated by VEP as high or moderate impact.

**Gene**	**Variant**	**HGVS**	**VarSome**	**Carriers**
**HDL deficiency, OMIM: 604091**				
*ABCA1*	chr9:107581932_G/A	ENSP00000363868.3:p.Pro1059Leu*	LP	3
**Dilated cardiomyopathy, OMIM: 302045 (XL)**				
*DMD*	chrX:32716075_G/A	ENSP00000354923.3:p.Ser291Phe*	VUS	5
**Hypertrophic cardiomyopathy**				
*ILK*	chr11:6625538_G/T	ENSP00000379975.2:p.Ala13Ser	VUS	3
*MYOM1*	chr18:3086053_T/A	ENSP00000348821.4:p.Thr1412Ser	VUS	3
*TRIM63*	chr1:26392766-26392799	N/A	N/A	7
*TRIM63*	chr1:26392801_A/T	ENSP00000363390.3:p.Leu97Gln	VUS	7
*TTN*	chr2:179455887_T/C	ENSP00000467141.1:p.Met20189Val	VUS	3
**Arrhythmogenic right ventricular dysplasia, OMIM: 600996 (AD)**				
*RYR2*	chr1:237777580_A/G	ENSP00000355533.2:p.Arg1718Gly*	LP	3
*TGFB3*	chr14:76429816_C/A	ENSP00000238682.3:p.Asp257Tyr	VUS	4
**Emery–Dreifuss muscular dystrophy, OMIM: 612998 (AD)**				
*SYNE1*	chr6:152665332_T/C	ENSP00000356224.5:p.Lys4037Glu*	VUS	3
*SYNE2*	chr14:64548225_C/A	ENSP00000350719.3:p.Thr3804Asn*	VUS	3
*SYNE2*	chr14:64606729_G/A	ENSP00000350719.3:p.Glu4972Lys*	VUS	3

*Genes are grouped based on associated disorders.*

*HGVS: variant description; strictly damaging missense variants are denoted with asterisk.*

*VarsSome: variant classification by VarSome according to the ACMG criteria.*

*P, pathogenic; LP, likely pathogenic; VUS, variant of unknown significance.*

*Carriers: number of individuals harboring the variant.*

The observed variant counts by type are tabulated for each gene in Supplementary Table 1. We found two genes that were significantly enriched with novel variants: *MIB1* associated with left ventricular non-compaction (OMIM: 615092), with 13 novel and 21 known variants, and *PHKA1* associated with muscle glycogenosis (OMIM:300559), with 12 novel and 17 known variants. The enrichment was validated by Fisher’s exact test that compared the numbers of novel and known variants in a particular gene vs. all genes taken together, with 1,356 novel and 10,520 known variants. The *P*-values for *MIB1* and *PHKA1* were 5.2 × 10^–5^ and 4.0 × 10^–5^, respectively. The next hit with excess of novel variants is *LAMA2* with 27 novel and 107 known variants; however, unlike the first two hits, its resulting *P* = 0.002 apparently does not pass any reasonable threshold even after the most lenient multiple test correction. Taking into account only rare known variants in comparison instead of all known variants gives similar results. The observed relative excess of rare variants may be a signature of population-specific demographic events at certain loci.

### ACMG59 Genes

Our sequencing target included 28 of the 59 genes known as the ACMG59 genes, for which the reporting of known pathogenic variants was recommended by the American College of Medical Genetics and Genomics (Kalia et al., 2017). Numerous studies reported the prevalence estimates for known and expected pathogenic variants in these genes (Amendola et al., 2015; Haer-Wigman et al., 2019; Kuo et al., 2020; Lacaze et al., 2020; Van Hout et al., 2020).

In the Ivanovo sample of 1,685 individuals, six genes (*DSP, KCNQ1, MYBPC3, MYH7, RYR2, TMEM43*) harbored eight known pathogenic or likely pathogenic variants (KP). Besides, nine novel high confidence protein-truncating variants were found in seven genes: *APOB, DSP, KCNQ1, MYH7, MYH11, PCSK9*, and *RYR2* (Table 3). All observed genotypes involving these variants were heterozygous, and none of the 17 variants were observed in the NWR exomes.

**TABLE 3:** Known pathogenic or likely pathogenic and novel protein-truncating variants in the 28 genes from the ACMG59 list.

**Gene**	**Variant**	**HGVS**	**VarSome**	**Carriers**
**Hypercholesterolemia, OMIM:144010 (AD); Hypobetalipoproteinemia, OMIM: 615558 (AR)**				
*APOB*	chr2:21232683_G/A	ENSP00000233242.1:p.Gln2353Ter	P	1
*APOB*	chr2:21234967_GA/G	ENSP00000233242.1:p.Phe1591SerfsTer19	P	1
*APOB*	chr2:21260870_AC/A	ENSP00000233242.1:p.Val166PhefsTer66	P	1
**Dilated cardiomyopathy, OMIM: 615821 (AD)**				
*DSP*	chr6:7580077_GACCA/G	ENSP00000369129.3:p.Thr1219ProfsTer30	P	1
*DSP*	rs121912997	ENSP00000369129.3:p.Arg1267Ter	P	1
**Long QT syndrome, OMIM: 192500 (AD)**				
*KCNQ1*	chr11:2466373_G/A	ENSP00000155840.2:p.Trp15Ter	P	1
*KCNQ1*	rs1337409061	ENSP00000155840.2:p.Thr96Arg	VUS	3
*KCNQ1*	rs199472814	ENSP00000155840.2:p.Arg591Leu	LP	1
*KCNQ1*	rs199473411	ENSP00000155840.2:p.Arg366Trp	P	1
**Hypertrophic cardiomyopathy, OMIM: 115197 (AD, AR), OMIM: 192600 (AD)**				
*MYBPC3*	rs376395543	ENST00000545968.1:c.26-2A > G	P	3
*MYH7*	chr14:23889261_CT/C	ENSP00000347507.3:p.Lys1173ArgfsTer41	P	2
*MYH7*	rs121913650	ENSP00000347507.3:p.Arg1712Trp	P	1
**Aortic aneurysm, OMIM: 132900 (AD)**				
*MYH11*	chr16:15917230_A/T	ENSP00000379616.3:p.Tyr128Ter	P	1
**Hypercholesterolemia; low level of LDL-C, OMIM: 603776 (AD)**				
*PCSK9*	chr1:55529040_A/T	ENST00000302118.5:c.1864-2A > T	VUS	1
**Arrhythmogenic right ventricular dysplasia, OMIM: 600996 (AD), 604400 (AD)**				
*RYR2*	chr1:237433817_G/GT	ENSP00000355533.2:p.Cys24LeufsTer61	P	1
*RYR2*	rs753850982	ENSP00000355533.2:p.Gly4095Ser	LP	1
*TMEM43*	rs63750743	ENSP00000303992.4:p.Ser358Leu	P	1

*Variant: dbSNP rsID for known variants or chr:pos_ref/alt identifier for novel PTVs.*

*HGVS: variant description.*

*VarSome: variant classification by VarSome according to the ACMG criteria.*

*P, pathogenic; LP, likely pathogenic; VUS, variant of unknown significance.*

*Carriers: number of Ivanovo individuals harboring the variant.*

We used VarSome (Kopanos et al., 2019) to classify KP and novel PTVs according to the ACMG criteria (Richards et al., 2015). All such variants in the ACMG59 genes were evaluated as pathogenic or likely pathogenic with two exceptions classified as variants of unknown significance (VUS). The first one is the missense substitution Thr96Arg in *KCNQ1* reported by one submitter as likely pathogenic without any supporting evidence and predicted as benign/tolerated by SIFT and PolyPhen-2. The second one is the novel splice acceptor variant in the *PCSK9* gene (ENST00000302118.5:c.1864-2A > T), considered high quality by LOFTEE and predicted deleterious by multiple tools. The LDL-C value of the carrier is 2.64 mmol/l which is in the normal range. In both cases, variants were classified by VarSome as VUS but not excluded from our analysis.

For all seven genes with novel PTVs, protein truncation is known to be an established disease-causing mechanism: in particular, no benign PTVs were reported in ClinVar for *KCNQ1*, *MYH11*, and *MYH7* and only one instance of benign PTV in *DSP*. In *PCSK9*, truncating mutations are associated with low plasma levels of low-density lipoprotein cholesterol (LDL-C) (Cohen et al., 2005). Six of eight known KPs were observed as singletons and 2 in 3 individuals each, giving 12 individuals in total which comprises 0.71% of the total sample of 1,685 participants. The initial set of variant calls included a KP variant in *RYR2* (rs794728721), but it was filtered out by both hg37 and hg38 GATK filters and was not included in the filtered variant set. Eight novel truncating variants were singletons, and one was observed in two participants, which gave the extra 10 participants harboring novel protein-truncating variants. The genes also harbored nine rare PTVs with worldwide maximal allele frequency below 0.1% as reported by VEP and the total of 14 carriers in the Ivanovo population (Supplementary Table 3). The 24 participants with novel or rare PTVs gave an extra 1.4% of the total sample of 1,685 participants. Taking in view our gene sequencing target included approximately one-half of the current list of 56 dominant ACMG59 genes (excluding *ATP7B*, *MUTYH*, and *PMS2*), one may approximate the prevalence of dominant KP variants and novel or rare PTVs in the Ivanovo population at 1.4 and 2.8%, respectively.

### All Genes

Evaluating the complete set of 242 sequenced genes, we identified 85 pathogenic or likely pathogenic ClinVar variants in 44 genes: 36 protein-truncating, 47 missense with 36 of them predicted to be strictly damaging, and two intron variants (Supplementary Table 3). This set of KP variants included eight variants in the ACMG59 genes described above. All observed genotypes involving known pathogenic or novel variants discussed in this section were heterozygous. Among the 77 known KP in non-ACMG59 genes, 9 and 63 were associated with dominant (AD) and recessive (AR) diseases, respectively (Table 4).

**TABLE 4:** Known pathogenic or likely pathogenic and novel protein-truncating variants in the full set of 242 targeted genes.

	**KP variants**			**Novel PTVs**		
**Genes**	**Variants**	**Genes**	**Carriers**	**Variants**	**Genes**	**Carriers**
ACMG (28)	8	6	12	9	7	10
Non-ACMG AD (78)	9	8	11	18	11	19
Non-ACMG AR (68)	63	26	124	20	16	20
Other (68)	5	4	13	22	15	22
Total	85	44		69	49	

*KP, known pathogenic or likely pathogenic variants from ClinVar.*

*PTV, protein-truncating variants.*

*The full set of targeted genes included 28 genes from the ACMG59 list; among the non-ACMG genes, 78 are dominant, 68 recessive, and 68 with other types of inheritance.*

Known variants in dominant non-ACMG genes were mostly present in one participant, except for the missense Pro279Leu variant in the *MEF2A* gene (rs121918529) observed in three participants and associated with CAD/myocardial infarction. As expected, known disease variants in recessive genes were more prevalent, with 12 recessive variants observed in three or more participants. Among them were two variants in *GAA* (rs375470378) and *PMM2* (rs28936415), found in eight participants each. One protein-truncating variant in *APOC3* without any assigned specific inheritance mode (rs138326449) was classified as pathogenic and associated with coronary artery disease and hyperalphalipoproteinemia by a single submitter in ClinVar (VCV000139560.3) and found in nine individuals.

The full set of 242 targeted genes also contained 69 novel PTVs in 48 genes (Table 4 and Supplementary Table 3), all being singletons, except for the two doubleton cases: the frameshift variant ENSP00000347507.3:p.Lys1173ArgfsTer41 in the ACMG59 gene *MYH7* and the stop gain variant ENSP00000364979.4:p.Arg1063Ter in the non-ACMG gene *COL4A1*. Unlike known pathogenic variants, novel PTVs did not exhibit any detectable enrichment or depletion in the dominant or recessive genes, suggesting that their functional role may require thorough evaluation.

We used VarSome (Kopanos et al., 2019) to classify KP and novel PTVs according to the ACMG criteria (Richards et al., 2015). All variants were evaluated as pathogenic or likely pathogenic with few exceptions: five KP variants and six novel PTVs were classified by VarSome as VUS, and one KP variant as likely benign (Supplementary Table 3). The latter is the missense Ala296Thr variant rs80356462 in the *SIX5* gene, classified by ClinVar as pathogenic, with no assertion criteria provided (ClinVar id: VCV000008599.1) and predicted benign by multiple computational tools, including SIFT and PolyPhen-2.

With novel PTVs observed in 48 genes, every fifth of the targeted genes in the Ivanovo sample contained a novel heterozygous PTV, although only in one or two individuals from the studied population. These genes included eight genes which are confidently depleted for PTV variation in gnomAD (Karczewski et al., 2020): *DSP, RYR2, COL4A1, EYA1, FBN2, MYH11, NNT*, and *SVEP1*. The most PTV-depleted gene in this list is the collagen type IV alpha 1 chain *COL4A1* with only six PTVs observed in gnomAD. The novel protein-truncating variant Arg1063Ter in this gene is observed in two participants.

We also detected 82 protein-truncating variants with worldwide maximal allele frequency below 0.1% as reported by VEP and refer to them as rare PTVs (Supplementary Table 3). Twenty-three of them (28%) are reported in ClinVar as VUS or variants with conflicting interpretation. Most of the rare PTV alleles are rare in the Ivanovo population as well, with only six seen in 3–6 individuals and one (rs201068740) in 14 individuals, all heterozygous.

### Overrepresented Known Pathogenic and Likely Pathogenic Variants

For the 16 KP variants harbored by three or more individuals, we compared the allele frequencies in our population with gnomAD v.2.1.1 non-Finnish Europeans (NFE). Since three or more copies of an allele correspond to population frequencies roughly equal or exceeding 0.1% in our sample with 1,685 individuals, we expected that KP variants observed in the Ivanovo population more frequently than in NFE would be present among such variants. Indeed, we found two dominant and eight recessive pathogenic variants with allele frequencies 6.7–67.2 times greater than in the NFE aggregated genomes and exomes allele counts (Table 5). In each case, the significance of the observed frequency difference was validated with the Fisher’s test on direct allele counts. The variants were accepted if the corresponding *P*-values did not exceed 0.05 after Benjamini–Hochberg correction (Supplementary Table 4).

**TABLE 5:** KP variants with frequencies in the Ivanovo population significantly exceeding the NFE.

**Gene, phenotype**	**Variant**	**HGVS**	**Carriers**	**Ivanovo/gnomAD ratio**
*KCNQ1*, Long QT syndrome (AD, 192500)	rs1337409061	ENSP00000155840.2:p.Thr96Arg	3	25.7
*MYBPC3*, Hypertrophic cardiomyopathy (AD, 115197)	rs376395543	ENST00000545968.1:c.26-2A > G	3	17.2
*GAA*, Glycogen storage disease (Pompe disease) (AR, 232300)	rs375470378	ENST00000302262.3:c.1552-3C > G	8	8.8
*GLB1*, GM1-gangliosidosis (AR, 253010, 230600)	rs376663785	ENSP00000306920.4:p.Tyr270Asp	4	25.4
*LAMA2*, Merosin-deficient congenital muscular dystrophy type 1A (AR, 607855)	rs398123387	ENST00000421865.2:c.7536del	4	67.2
*MTO1*, Combined oxidative phosphorylation deficiency (AR, 614702)	rs201544686	ENSP00000402038.2:p.Arg517His	6	7.7
*SURF1*, Mitochondrial complex IV deficiency, Leigh syndrome (AR, 220110)	rs782316919	ENST00000371974.3:c.845_846del	4	8.0
*ALMS1*, Alstrom syndrome (AR, 203800)	rs797045228	ENST00000264448.6:c.4150dup	3	19.0
*ALMS1*, Alstrom syndrome (AR, 203800)	rs747272625	ENST00000264448.6: c.11310_11313del	3	16.7
*SCO2*, Cardioencephalo-myopathy (AR, 604377)	rs74315511	ENSP00000444433.1: p.Glu140Lys	4	6.7

*Carriers: number of Ivanovo individuals harboring the variant.*

*Ivanovo/gnomAD is the ratio of allele frequencies in our sample and gnomAD NFE genomes and exomes considered together.*

Missense substitution Thr96Arg in *KCNQ1* (dbSNP: rs1337409061) was recently submitted to ClinVar as likely pathogenic and associated with long QT syndrome (ClinVar id: VCV000983021.1). This allele was observed in four copies only in non-Finnish Europeans with the frequency of 3.4 × 10^–5^ in that population. With three harboring participants, the frequency in the Ivanovo population equals 8.9 × 10^–4^ with lower and upper 95% confidence interval (CI) margins 2.0 × 10^–4^ and 2.6 × 10^–3^, respectively. The Thr96Arg substitution, however, was classified by VarSome as VUS, had no supporting evidence for pathogenicity in ClinVar, and was not observed among the variants discovered in 9 and 53 unrelated Russian families with the long QT syndrome (Polyak et al., 2016; Maltese et al., 2017). The presence of this variant in the general Russian population without any prevalence in patients may suggest that its pathogenicity needs to be confirmed by future independent submissions.

Pathogenic splice acceptor variant c.26-2A > G in *MYBPC3* (rs376395543) is associated with hypertrophic cardiomyopathy (VCV000042644.10) and is observed in six copies in non-Finnish Europeans (frequency 5.2 × 10^–5^) and one allele copy in Latino Americans. This variant was observed in three individuals in our set, with population frequency estimated as 8.9 × 10^–4^ (CI from 2.0 × 10^–4^ to 2.6 × 10^–3^), not observed in NWR exomes and classified as pathogenic by VarSome.

Table 5 also contains eight pathogenic recessive variants which were significantly overrepresented in the Ivanovo population compared to gnomAD NFE. The most frequent one harbored by eight participants in our study was the acid alpha-glucosidase *GAA* intronic variant c.1552-3C > G (rs375470378) associated with glycogen storage disease type II, also known as Pompe disease. This intronic variant is reported in ClinVar as pathogenic or likely pathogenic by multiple submitters (VCV000419722.9) and likely pathogenic by VarSome. The observed frequency in the Ivanovo population (0.24%) significantly exceeds the NFE (0.03%) and other population frequencies, with the exception of the Estonian population where the observed frequency is 3/4480 (0.07%) with marginally significant difference from the Ivanovo population (Fisher’s test *P* = 0.045) likely due to modest study sample sizes. The northwest Russia frequency is estimated as 0.14%, suggesting that this variant is mostly prevalent in Northern Europe. Glycogen storage disease type II (Pompe disease) is an autosomal recessive metabolic disorder which damages muscle and nerve cells and results from the accumulation of glycogen in the lysosome due to the deficiency of the alpha-glucosidase enzyme. The worldwide prevalence of this disease is in the range 1:14,000 to 1:300,000 (Van der Ploeg and Reuser, 2008), with no estimates for Russia available (Semyachkina et al., 2014). It was also hypothesized that the overwhelming majority of Pompe disease cases in Russia may not be diagnosed (Nikitin, 2016). By combining allele counts in our study and the NWR, we calculated the frequency of homozygous carriers of the disease-causing *GAA* variant rs375470378 as 1:226,000 which is the lower estimate of Pompe disease prevalence in Russia due to the existence of other pathogenic alleles.

The largest frequency difference between NFE and our sample was observed for the alpha-2 laminin *LAMA2* frameshift deletion c.7536delC (rs398123387) associated with merosin-deficient congenital muscular dystrophy type 1A (MDC1A) and segregating in the Ivanovo population in four copies (0.11%), which is approximately 67 times greater than the NFE frequency of 0.0017%. This variant was reported earlier in a single Russian patient (Dadali et al., 2010) and later in 21% of all affected chromosomes in the sample of 29 unrelated MDC1A patients, suggesting that this is the most abundant disease-causing *LAMA2* allele in the Russian population (Milovidova et al., 2018). Based on the analysis of four microsatellite markers in the *LAMA2* region of chromosome 6, the authors suggested that this mutation belongs to a founder haplotype spanning at least 3.2 cM. Our data seem to confirm this assumption and suggest the frequency range (0.11% with CI from 0.03 to 0.30%) for this variant in the unaffected population.

The frameshift variant c.845_846del in the Cytochrome C oxidase assembly Factor *SURF1* (rs782316919) is associated with Leigh syndrome due to mitochondrial complex IV deficiency (VCV000012770.12) and has been reported earlier as the most prevalent *SURF1* disease allele among Russian, Ukrainian, and Polish patients (Piekutowska-Abramczuk et al., 2009; Tsygankova et al., 2010). Our frequency estimate of 0.11% is comparable with those given earlier for the NWR and Poland (0.22 and 0.28%, respectively) and is by order of magnitude higher than that in the NFE (0.014%), confirming the hypothesis of the Eastern European origin of this variant (Piekutowska-Abramczuk et al., 2009).

The missense variant Arg517His in the *MTO1* gene associated with combined oxidative phosphorylation deficiency (rs201544686, VCV000089037.4) and c.4150dup duplication in the *ALMS1* gene associated with the Alstrom syndrome (rs797045228, VCV000210127.8) were reported earlier among 14 known pathogenic variants most prevalent in NWR (Barbitoff et al., 2019). It is noteworthy that we discovered another pathogenic variant in *ALMS1* (rs747272625, VCV000550797.4) detected in three other participants and overrepresented in the Ivanovo population compared to NFE (0.09% vs. 0.005%). The missense Tyr270Asp variant in *GLB1* is associated with GM1-gangliosidosis (rs376663785, VCV000284172.5) and was observed in three copies in the Ivanovo population. Finally, the missense substitution Glu140Lys in *SCO2* is associated with cardioencephalomyopathy, also known as mitochondrial complex IV deficiency (rs74315511, VCV000005681.11). The observed Ivanovo frequency for this allele (0.0012) is 6.7 greater than the overall NFE frequency (0.00017) and most close to that of the Estonian subset of NFE (0.0008) and NWR (0.0007), suggesting the putative geographical origin of this variant.

### Phenotype Evaluation

We analyzed the available clinical data of participants with detected pathogenic/likely pathogenic variants or novel protein-truncating variants in 28 of ACMG59 genes and other genes with autosomal dominant inheritance. For the ACMG59 genes, we also evaluated phenotypes in the carriers of rare protein-truncating variants (VEP MAX_AF < 0.1%). Variants subject to carrier evaluation are marked in Supplementary Table 3; the results are presented in Table 6. For the first group that contained 17 KP variants in 23 carriers, we found five individuals with matching phenotypes: prolonged QTc interval (Arg366Trp in *KCNQ1* and Arg858His in *CACNA1C*), hypertriglyceridemia (Gln313Ter in *APOA5*), CAD (Pro279Leu in *MEF2A*), and cerebral infarction (Arg153Cys in *NOTCH3*).

**TABLE 6:** Variants with confirmed phenotypes.

**Gene**	**ACMG**	**Variant**	**HGVS**	**Phenotype (Source)**
**I. Known pathogenic or likely pathogenic: 17 variants, 23 carriers**				
*KCNQ1*	Yes	rs199473411 VCV000052955.8	ENSP00000155840.2: p.Arg366Trp	Prolonged QTc interval, QT = 453 mc for male (ECG)
*CACNA1C*	No	rs786205753 VCV000190653.57	ENSP00000266376.6: p.Arg858His	Prolonged QTc interval, QT = 453 mc for female (ECG)
*APOA5*	No	rs147528707 VCV000420172.2	ENSP00000445002.1: p.Gln313Ter	Hypertriglyceridemia, TG = 14.3 mmol/l (Biochemical assay)
*MEF2A*	No	rs121918529 VCV000008949.1	ENSP00000346389.5: p.Pro279Leu	CAD (CAD validation)
*NOTCH3*	No	rs797045014 VCV000208501.2	ENSP00000263388.1: p.Arg153Cys	Cerebral infarction (Endpoint)
**II. Novel protein truncating: 27 variants, 27 carriers**				
*APOB*	Yes	chr2:21232683_G/A	ENSP00000233242.1: p.Gln2353Ter	Hypobetalipoproteinemia, LDL-Ñ = 1.47 mmol/l (Biochemical assay)
*APOB*	Yes	chr2:21234967_GA/G	ENSP00000233242.1: p.Phe1591SerfsTer19	Hypobetalipoproteinemia, LDL-Ñ = 0.95 mmol/l (Biochemical assay)
*APOB*	Yes	chr2:21260870_AC/A	ENSP00000233242.1: p.Val166PhefsTer66	Hypobetalipoproteinemia, LDL-Ñ = 0.72 mmol/l (Biochemical assay)
*MYH7*	Yes	chr14:23889261_CT/C	ENSP00000347507.3: p.Lys1173ArgfsTer41	Hypertrophic cardiomyopathy (Medical record)
**III. Rare protein truncating: 9 variants, 14 carriers (ACMG59 genes only)**				
*RYR1*	Yes	rs797045932 VCV000212099.3	ENSP00000352608.2: p.Pro836LeufsTer48	Myopathy (Medical record)
*KCNQ1*	Yes	rs397508104 VCV000053027.10	ENSP00000155840.2: p.Arg632GlnfsTer20	Prolonged QTc interval, QT = 458 mc for female (ECG)

*Variant: dbSNP rsID for known variants or chr:pos_ref/alt identifier for novel PTVs.*

*HGVS: variant description.*

*Phenotype: disease phenotype confirmed by evaluation of clinical data; source of clinical data is specified in the parentheses.*

The second group, novel PTVs, comprised 27 variants in 27 carriers and yielded four individuals: three with hypobetalipoproteinemia, all with various novel truncating mutations in *APOB*, and one individual with hypertrophic cardiomyopathy and frameshift deletion in *MYH7* (Table 6). Various truncated forms of *APOB* have been found earlier to segregate with the familial hypobetalipoproteinemia phenotype (OMIM:615558). Missense mutations are the most prevalent cardiomyopathy-related pathogenic mechanism in *MYH7*; however, ClinVar reports 20 cases of pathogenic and likely pathogenic truncating variants with various degrees of confidence.

Finally, nine rare PTVs in the ACMG59 genes were harbored by 14 carriers, two of which displayed expected phenotypes: myopathy (frameshift deletion in *RYR1*) and prolonged QTc interval (frameshift insertion in *KCNQ1*). We also checked 17 carriers of 18 novel truncating variants in non-ACMG59 genes and did not discover any remarkable genotype–phenotype associations.

Out of the total 11 genotype–phenotype associations described above, 7 are in the ACMG59 genes: 1 among KP variants, 4 among novel PTVs, and 2 among the rare PTVs. Taking in view that the total numbers of carriers checked in these three groups were 12, 10, and 14, respectively (Supplementary Table 3), we estimated the overall success rate of phenotype evaluation in the ACMG59 genes as 19% (=7/36). It is worth noting that the highest phenotype confirmation rate (40%) was observed in the subset of novel PTVs, thus emphasizing the importance of studying this class of variation by deeper sequencing the individuals from the Russian population.

In the course of the analysis of the study endpoints, we found a female participant who died of heart failure due to dilated cardiomyopathy. The manual review of rare candidate variants harbored by this individual discovered the singleton missense variant Thr891Met in the *FLNC* gene (rs766023596). This substitution was submitted to ClinVar (VCV000855393.2) by a single submitter as VUS associated with this type of cardiomyopathy (Cui et al., 2018). Our observation provides evidence suggesting the pathogenicity of this variant.

## Discussion

The overwhelming majority of variants detected in our study in three or more copies were known and shared with other populations. The alternative allele frequencies of these variants were highly correlated with non-Finnish European population. This is in agreement with the earlier observation that in the case of a smaller sample set of 694 individuals from the northwest region of Russia, the majority of disease alleles were shared between Russian and European populations (Barbitoff et al., 2019). However, with the sample size of 1,685 individuals, we detected a considerable fraction (11.4%) of novel variants not present in the dbSNP and not reported earlier in the Russian population. Most such variants were singletons and doubletons in our sample, with the exceptions discussed above: only 26 novel alleles were observed in three-to-seven copies, with no protein-truncating variants among them. Our results therefore suggest that virtually no “private” alleles specific for the Russian population reach the frequencies above 0.1–0.2%

The fraction of novel variants was highest among the protein-truncating ones: out of 218 PTVs discovered in our dataset, 70 are novel and, with one exception, not reported before in the Russian population. This effect can be explained by the purifying selection acting on specific classes of variants enriched with deleterious alleles. Since rare and novel protein-truncating variants are of special interest in the context of clinical genome interpretation (DeBoever et al., 2018), our study emphasizes the importance of deeper sequencing, taking in view the size and ethnic diversity of the Russian population.

Approximately 18% of 242 targeted genes harbored known pathogenic or likely pathogenic variants reported earlier in ClinVar. Similar to the novel variants, most of disease-causing alleles were singletons or doubletons in our sample. We report nine variants with disease alleles significantly more frequent in the Ivanovo population compared to the non-Finnish Europeans. The largest frequency difference between NFE and our sample (>67x) was observed for the alpha-2 laminin *LAMA2* frameshift deletion c.7536delC (rs398123387) associated with merosin-deficient congenital muscular dystrophy type 1A (MDC1A). It was suggested earlier that this deletion is the most abundant disease-causing *LAMA2* mutation in the Russian population residing on the founder haplotype spanning at least 3.2 cM (Milovidova et al., 2018). We estimated the frequency range for this variant in the unaffected population as 0.11 with 95% CI range from 0.03 to 0.30%.

To the best of our knowledge, this is the first report of the spectrum of known and putative pathogenic variants in the ACMG59 genes in the Russian population. We approximated the prevalence of known dominant pathogenic variants as 1.4% which is in agreement with the range of 1.3−2.7% suggested recently for other European populations (Haer-Wigman et al., 2019; Kuo et al., 2020; Lacaze et al., 2020; Van Hout et al., 2020). Since our target included only 28 genes of the ACMG59 list, further studies may provide more precise estimates of KP variant prevalence in these genes in the Russian population. Our results also emphasize the importance of sequencing representative cohorts (>1,000 individuals) to uncover the population-specific genetic variation of clinical relevance.

We observed the incomplete penetrance of known pathogenic variants in the ACMG59 genes: only 1 individual out of 12 with KP variants in these genes had the phenotype most likely related to the variant. When known pathogenic and novel or rare protein-truncating variants were considered together, the overall rate of confirmed phenotypes was about 19%, with maximum in the subset of novel PTVs. This is in overall agreement with the other studies: in the population cohort from the Rotterdam Study, there were 13% of the carriers of known pathogenic variants in the ACMG59 genes who had phenotype correlated with genotype (Van Rooij et al., 2020). The Rotterdam study was based on a larger sample size of 2,628 individuals and involved an in-depth analysis of the phenotypes. The Rotterdam study included persons of age 55 and above, while in our study, the age of participants was above 25, which enabled the identification of individuals with earlier disease onset.

Hou et al. (2020) reported results of the whole-genome sequencing of 1,190 self-referred volunteers with a median age of 54 years (range 20–89+ years, 70.6% European) and showed that deeper phenotyping (metabolomics, advanced imaging, and clinical laboratory tests in addition to family/medical history) significantly increased the concordance between genetic and phenotypic data. Genotype–phenotype associations were identified in 66.5% of the participants with pathogenic or likely pathogenic variants, not restricted to the ACMG59 genes. Overall, 44.5% of the cases were revealed through genomics and metabolomics analysis and had phenotype manifestations affecting serum metabolite levels. We hypothesize that the volume of the available clinical information is the limiting factor for our ability to observe better concordance between genotype and phenotype. In particular, results of imaging tests, which are essential components of cardiomyopathy diagnostics, were not available for all Ivanovo participants. The medical records, which usually contain more detailed clinical information about a patient, were very useful in our analysis and enabled identification of two genotype–phenotype associations in 10 variant carriers with available medical records. In order to develop a procedure for the return of secondary findings in the ACMG59 genes, we are designing an in-depth clinical examination targeted at all study participants with detected known and expected pathogenic variants in these genes and the genetic cascade screening of their families.

This work presents the analysis of targeted sequencing of the largest sample of the general Russian population to date. The observed spectrum of genetic variants in a region with predominantly Russian population is close in genetic composition to European populations, with the exception of a rare variation. We believe that our results provide a valuable reference for the clinical interpretation of genome sequencing and are the first step toward creating a comprehensive reference of genetic variability observed in the Russian Federation.

## Data Availability Statement

Aggregated variant frequency information can be requested from the authors. Individual genotype information cannot be made available in order to protect participant privacy.

## Ethics Statement

The studies involving human participants were reviewed and approved by the independent Ethics Committee of the National Medical Research Center for Therapy and Preventive Medicine (Protocol numbers 07-03/12 from 03.07.2012). The patients/participants provided their written informed consent to participate in this study.

## Author Contributions

OD, AM, AE, SS, and VR conceived and designed the study. VR, AE, AZ, MZ, AK, and YV analyzed the data and wrote the manuscript. IE, GM, OB, SR, and MP carried out participant and clinical data management. AK, ES, OK, OS, and MD performed the target design and sequencing. All authors contributed to the article, revised the manuscript, and approved the submitted version.

## Conflict of Interest

The authors declare that the research was conducted in the absence of any commercial or financial relationships that could be construed as a potential conflict of interest.

## Publisher’s Note

All claims expressed in this article are solely those of the authors and do not necessarily represent those of their affiliated organizations, or those of the publisher, the editors and the reviewers. Any product that may be evaluated in this article, or claim that may be made by its manufacturer, is not guaranteed or endorsed by the publisher.
