# Supplementary material for: Targeted Sequencing of 242 Clinically Important Genes in the Russian Population From the Ivanovo Region
Source: Front Genet. 2021 Oct 7;12:709419. doi: 10.3389/fgene.2021.709419 (PMC8529250; doi:10.3389/fgene.2021.709419)
Supplement: Supplementary file 1 [file Data_Sheet_1.docx]

Supplementary Material

# Supplementary Table Captions

**Supplementary Table 1.** Overview of 242 targeted genes. Columns:

Variant and carrier counts for each gene. Path: KP variants; VUS: variants of unknown significance; PTV: protein-truncating variants; MisSDam: missense strictly damaging; MisOther: other missesne variants; Inframe: inframe indels; Other, UTR, intron, synonymous etc. variants. ACMG59: genes from ACMG59 gene list; oe_syn_upper, oe_mis_upper, oe_lof_upper: gene constraint metrics based on variant counts in gnomAD (Karczewski et al., 2020). Inheritance: inheritance model; Phenotype: associated disorders with OMIM identifier (if applicable).

**Supplementary Table 2.** Novel variants with allele count AC>2. Columns:

| 1 | #Chr | Chromosome |
| --- | --- | --- |
| 2 | Pos | Position |
| 3 | Variant | Variant id |
| 4 | Ref | Reference allele |
| 5 | Alt | Alternative allele |
| 6 | Feature | Transcript id (VEP) |
| 7 | Consequence | Consequence type: missense, splice_acceptor_variant, etc. (VEP) |
| 8 | Existing_variation | dbSNP id (VEP) |
| 9 | IMPACT | Subjective impact classification of consequence type (VEP) |
| 10 | LoF | Quality prediction for high impact variants: HC=High Quality, LC=Low Quality, OS=other splice variants (VEP, LOFTEE plugin) |
| 11 | SIFT | Effect prediction for missenses |
| 12 | PolyPhen | Effect prediction for missenses |
| 13 | STRAND | Transcript strand (VEP) |
| 14 | VARIANT_CLASS | SNV/insertion/deletion |
| 15 | SYMBOL | Gene symbol name (HGNC, etc.) (VEP) |
| 16 | BIOTYPE | Biotype of transcript or regulatory feature (VEP) |
| 17 | ACMG59 | ACMG59/other |
| 18 | CANONICAL | Indicates if transcript is canonical for this gene: YES/- (VEP) |
| 19 | HGVSc | HGVS coding sequence name (VEP) |
| 20 | HGVSp | HGVS protein sequence name (VEP) |
| 21 | VarSome | VarSome classification: P, pathogenic; LP, likely pathogenic; VUS: variant of unknown significance; LB, likely benign; B, benign |
| 22 | 1000G.AF | Alt.allele frequency in 1000 Genomes combined population (VEP) |
| 23 | MAX_AF_POPS | Populations in which maximum frequency was observed (VEP) |
| 24 | MAX_AF | Maximum alt.allele frequency in 1000 Genomes, ESP and ExAC/gnomAD (VEP) |
| 25 | gnomADe.AF | Alt.allele frequency in gnomAD exomes combined population (gnomAD exomes) |
| 26 | gnomADe.popmax | GnomAD exomes population in which maximum frequency was observed (gnomAD exomes) |
| 27 | gnomADe.AF_popmax | Maximum alt.allele frequency in gnomAD exomes populations (gnomAD exomes) |
| 28 | gnomADe.AF_nfe | Alt.allele frequency in European non-Finnish gnomAD exomes (gnomAD exomes) |
| 29 | gnomADg.AF | Alt.allele frequency in gnomAD exomes combined population (gnomAD exomes) |
| 30 | gnomADg.popmax | GnomAD exomes population in which maximum frequency was observed (gnomAD exomes) |
| 31 | gnomADg.AF_popmax | Maximum alt.allele frequency in gnomAD exomes populations (gnomAD exomes) |
| 32 | gnomADg.AF_nfe | Alt.allele frequency in European non-Finnish gnomAD exomes (gnomAD exomes) |
| 33 | ClinSigFull | Clinical significance (ClinVar) |
| 34 | ClinSigShort | Simplified ClinSig: Pathogenic, Benign, VUS (ClinVar), Conflict |
| 35 | PhenIDS | Phenotype IDs (ClinVar) |
| 36 | PhenList | Phenotype list (ClinVar) |
| 37 | Origin | Origin: somatic/germline (ClinVar) |
| 38 | ReviewStatus | Review status (ClinVar) |
| 39 | NumSubmitters | Number of submitters (ClinVar) |
| 40 | Inheritance | Inheritance (MedGen) |
| 41 | NWR.AF | AF in 694 NorthWest regions (Barbitoff 2019) |
| 42 | NWR.AC | AC in 694 NorthWest regions (Barbitoff 2019) |
| 43 | NWR.AN | AN in 694 NorthWest regions (Barbitoff 2019) |
| 44 | QUAL | Variant quality (GATK) |
| 45 | FILTER.hg37 | Variant filter with hg37 reference (GATK) |
| 46 | FILTER.hg38 | Variant filter with hg38 reference (GATK) |
| 47 | AF | Allele frequency in our sample (GATK) |
| 48 | AC | Allele count in genotypes (GATK) |
| 49 | AN | Total number of alleles in called genotypes (GATK) |
| 50 | NumHomAlt | Number of homalt samples (bcftools) |
| 51 | Region | Low complexity genome region? (SDUST) |
| 52 | MultiSite | Multiallelic site? |
| 53 | MaxGQ | Maximum GQ among alt. allele carriers (GATK) |
| 54 | AveGQ | Average GQ among alt. allele carriers (GATK) |
| 55 | Phenotype evaluation | Variant selected for phenotype evaluation of alt. allele carriers |

**Supplementary Table 3.** Known pathogenic or likely pathogenic, novel or rare protein truncating variants. Columns are as in Supplementary Table 2.

**Supplementary Table 4.** Known pathogenic variants with frequencies in the Ivanovo population significantly exceeding the non-Finnish Europeans; extended version of Table 5.

Variant, dbSNP rsID; HGVS: variant description; VarSome, automated variant classification according to the ACMG guidelines; gnomAD, allele frequenct in gnomAD NFE genomes and exomes considered together; Ivanovo AC, allele counts in the Ivanovo population; Ivanovo AF, allele counts in the Ivanovo population; Ivanovo/gnomAD is the ratio of allele frequencies in our sample and gnomAD NFE genomes and exomes considered together; P-value, Fisher test on direct allele counts, raw and Bonferroni corrected; CI for Ivanovo AF; NWR, variant frequency in 694 northwest exomes (Barbitoff et al., 2019).

# Selection of participants

The multi-stage clustered sample was obtained using Kish methods (Kish, 1965). District outpatient departments (polyclinics) were randomly selected as primary sampling units (PSU), each from 30,000 to 80,000 adult inhabitants. Then, five sites (physician’s localities) from every polyclinic were randomly selected as a secondary sampling unit (SSU), which consisted of approximately 2000 adult people. On each site, 100 households were selected as tertiary sampling units (TSU) and in every household an available study subject was found. One PSU in selected regions was rural while all the others were urban. The total sample size was calculated using the formula: 4 PSU × 5 SSU × 100 TSU = 2000 subjects, selected to be observed in the region with intended response rate of 80%. Blood samples of all individuals were stored at −70 °C at the Biobank of the National Medical Research Center for Therapy and Preventive Medicine (Moscow, Russia).

# Target panel sequencing

The isolation of DNA was done using the QIAamp DNA Blood Mini Kit (Qiagen, Hilden, Germany). DNA concentration was assessed with a Qubit 4.0 fluorimeter (Thermo Fisher Scientific, Waltham, MA, USA). The library preparation was done using the SeqCap EZ Prime Choice Library kit (Roche, Basel, Switzerland). All stages of sequencing were performed using Mid Output (paired-end, 2x150 bp, average insert size 269 bp) according to the manufacturers’ protocols with Nextseq 550 (Illumina, San Diego, CA, USA). Overall, targeted regions were sequenced to an average depth of 154 bp, and 99% of the coding sequence for these genes was covered at least 40-fold. With this coverage depth, 88.1% of genotypes are called with maximal genotype quality GQ of 99. Sanger sequencing was also used to validate NGS results for selected variants.

# Variant confirmation by Sanger sequencing

Selected variants of interest were validated using Sanger sequencing. PCRs were performed in 20 μL of a mixture containing 0.2 mM of each nucleotide, 1× PCR buffer, 20 ng of the DNA, 10 ng of each primer, 2.5 U of DNA polymerase. Amplification was performed on a GeneAmp PCR System 9700 thermocycler (Thermo Fisher Scientific, Waltham, MA, USA) with the following parameters: 95 °C—300 s; 30 cycles: 95 °C—30 s, 62 °C—30 s, 72 °C—30 s; 72 °C—600 s. The amplicons were purified using ExoSAP-IT (Affymetrix, Santa Clara, CA, USA). The nucleotide sequence of PCR products was determined using the ABI PRISM BigDye Terminator reagent kit v. 3.1 (Thermo Fisher Scientific, Waltham, MA, USA) followed by analysis of the reaction products on an automated DNA sequencer Applied Biosystem 3500 DNA Analyzer (Thermo Fisher Scientific, Waltham, MA, USA). All steps of Sanger sequencing were done in accordance with the manufacturer’s recommendations.

# Bioinformatic pipeline

Paired-end raw sequencing data were converted to pairs of fastq files with bcl2fastq v.2.20.0.422. Initial quality assessment of reads was performed with FastQC software v.0.11.8 (Andrews et al., 2010). Paired-end reads were then trimmed with Trimmomatic v.0.39 (Bolger et al., 2014) from the 3' end to remove low-quality bases with Phred Quality Score below 20 (TRAILING:20) to a minimum allowed length of 25 nt. Reads were mapped to the human reference genome with bwa mem (v.0.7.17-r1188, human reference hg19) with output in BAM format (Li, H. and Durbin, 2009). Picard v. 2.20.0 (Broad Institute, 2018) was employed to mark optical and PCR duplicates (MarkDuplicates), and to label read group fields appropriately, sort and index the BAM file (AddOrReplaceReadGroups).

For sample quality control we reported and controlled numerous metrics, in particular, the total number of reads per sample, the optical and PCR duplicate rate, the total number of reads mapped to the target regions. Number of total PE reads was minimum 2.2 mln with the mean of 3.4 mln per sample. After marking duplicates, there were at least 1.2 mln unique PE reads per sample. On average 57% of reads were mapped to the target. We used Cleancall (Flickinger et al., 2015) with default settings to detect within-species DNA sample contamination.

GATK (v.3.8-1-0-gf15c1c3ef) (Poplin et al., 2017) modules RealignerTargetCreator and IndelRealigner were used to find intervals for potential realignment and to realign reads around indels. Genome-wide variant calling was performed in each sample by GATK3 module HaplotypeCaller with output in gVCF format.

To speed up subsequent gVCF file processing, the reference human genome was split into 110 non-overlapping genomic intervals. For each interval all individual gVCF files were merged into a single gVCF file using GATK CombineGVCFs tool. The combined gVCF was converted to a merged VCF file with GATK GenotypeGVCF. Indels in the merged VCF file were left aligned using bcftools norm (v1.11) tool, posterior genotype refinement performed with GATK CalculateGenotypePosteriors assuming unrelated samples.

The refined callset was split into SNP and Indel parts with hard filters applied separately to each subset. SNPs were filtered out if any of the conditions was met:

- MQ (root mean square mapping quality) < 40.0
- QD (quality by depth) < 2.0
- FS (Phred-scaled probability of strand bias) > 60.0
- SOR (symmetric odds ratio) > 3.0
- MQRankSum (mapping quality rank sum) < -12.5
- ReadPosRankSum (read position rank sum) < -8.0
- ExcessHet (Phred-scaled p-value for exact test of excess heterozygosity) > 54.69
- QUAL (Phred-scaled variant quality) < 50.0

Similarly, Indels were filtered out according to the following conditions:

- QD (quality by depth) < 2.0
- FS (Phred-scaled probability of strand bias) > 200.0
- ReadPosRankSum (read position rank sum) < -20.0
- ExcessHet (Phred-scaled p-value for exact test of excess heterozygosity) > 54.69
- QUAL (Phred-scaled quality) < 50.0

All threshold values were adopted from GATK Best Practices for hard filtering and recommended values, except for the increased threshold for the QUAL filter.

Filtered SNPs and indels were combined for each genome interval back to a single VCF file with bcftools concat; the final callset was constructed by concatenating individual interval files and then restricting the resulting single file to the target genome intervals. Variant Quality Score Recalibration (VQSR) filters were implemented for SNPs and indels with GATK VariantRecalibrator and ApplyRecalibration tools. The tranche sensitivity threshold for both SNP and Indels was selected as 99.5%, for the indels the maxGaussian parameter was lowered to 3 due to the low number of indels in the training set. The acquired VQSR filters were joined with hard filters for the same variants.

Genome low complexity regions were identified with SDUST (Morgulis et al., 2006) at a score threshold of 30 following gnomAD practice. Variants in low complexity regions were marked with LowCompl flag. Heterozygous genotypes at multiallelic sites not involving reference allele (1/2, 2/3, etc) were set to unknown. Finally, allele counts and variant population frequencies (AN, AF, AC fields) were recalculated, variants without alternative alleles (lost e.g. due to genotype refinement) were removed, and multiallelic variants were split into biallelic variants using bcftools norm tool. This procedure generates the final VCF file with variant and genotype calls.

# Supplementary references

Andrews, S. (2010). FastQC: A Quality Control Tool for High Throughput Sequence Data. Available at: http://www.bioinformatics.babraham.ac.uk/projects/fastqc/%0A.

Bolger, A. M., Lohse, M., and Usadel, B. (2014). Trimmomatic: a flexible trimmer for Illumina sequence data. *Bioinformatics* 30, 2114–2120. doi:10.1093/bioinformatics/btu170.

Broad Institute, G. R. (2018). Picard Toolkit. Available at: http://broadinstitute.github.io/picard/.

Flickinger, M., Jun, G., Abecasis, G. R., Boehnke, M., and Kang, H. M. (2015). Correcting for Sample Contamination in Genotype Calling of DNA Sequence Data. *Am. J. Hum. Genet.* 97, 284–290. doi:10.1016/j.ajhg.2015.07.002.

Kish, L. (1965). Survey Sampling. *John Wiley Sons*.

Li, H., and Durbin, R. (2009). Fast and accurate short read alignment with Burrows-Wheeler transform. *Bioinformatics* 25, 1754–1760. doi:10.1093/bioinformatics/btp324.

Morgulis, A., Gertz, E. M., Schäffer, A. A., and Agarwala, R. (2006). A Fast and Symmetric DUST Implementation to Mask Low-Complexity DNA Sequences. *J. Comput. Biol.* 13, 1028–1040. doi:10.1089/cmb.2006.13.1028.

Poplin, R., Ruano-Rubio, V., DePristo, M., Fennell, T., Carneiro, M., Van der Auwera, G., et al. (2017). Scaling accurate genetic variant discovery to tens of thousands of samples. *bioRxiv*. doi:10.1101/201178.
